# Functional Insights into the Kelp Microbiome from Metagenome-Assembled Genomes

**DOI:** 10.1128/msystems.01422-21

**Published:** 2022-06-01

**Authors:** Brooke L. Weigel, Khashiff K. Miranda, Emily C. Fogarty, Andrea R. Watson, Catherine A. Pfister

**Affiliations:** a Committee on Evolutionary Biology, University of Chicago, Chicago, Illinois, USA; b The College, University of Chicago, Chicago, Illinois, USA; c Committee on Microbiology, University of Chicago, Chicago, Illinois, USA; d Department of Medicine, University of Chicago, Chicago, Illinois, USA; e Department of Ecology & Evolution, University of Chicago, Chicago, Illinois, USA; University of Massachusetts Amherst

**Keywords:** *Granulosicoccus*, alginate, dissolved organic matter, host-microbe, kelp, metagenome-assembled genomes, metagenomics, microbiome, nitrogen cycling, vitamin B_12_

## Abstract

Eukaryotic organisms evolved in a microbial world and often have intimate associations with diverse bacterial groups. Kelp, brown macroalgae in the order *Laminariales*, play a vital role in coastal ecosystems, yet we know little about the functional role of the microbial symbionts that cover their photosynthetic surfaces. Here, we reconstructed 79 bacterial metagenome-assembled genomes (MAGs) from blades of the bull kelp, Nereocystis luetkeana, allowing us to determine their metabolic potential and functional roles. Despite the annual life history of bull kelp, nearly half of the bacterial MAGs were detected across multiple years. Diverse members of the kelp microbiome, spanning 6 bacterial phyla, contained genes for transporting and assimilating dissolved organic matter (DOM), which is secreted by kelp in large quantities and likely fuels the metabolism of these heterotrophic bacteria. Bacterial genomes also contained alginate lyase and biosynthesis genes, involved in polysaccharide degradation and biofilm formation, respectively. Kelp-associated bacterial genomes contained genes for dissimilatory nitrate reduction and urea hydrolysis, likely providing a reduced source of nitrogen to the host kelp. The genome of the most abundant member of the kelp microbiome and common macroalgal symbiont, *Granulosicoccus*, contained a full suite of genes for synthesizing cobalamin (vitamin B_12_), suggesting that kelp-associated bacteria have the potential to provide their host kelp with vitamins. Finally, kelp-associated *Granulosicoccus* contained genes that typify the aerobic anoxygenic phototrophic bacteria, including genes for bacteriochlorophyll synthesis and photosystem II reaction center proteins, making them the first known photoheterotrophic representatives of this genus.

**IMPORTANCE** Kelp (brown algae in the order *Laminariales*) are foundational species that create essential habitat in temperate and arctic coastal marine ecosystems. These photosynthetic giants host millions of microbial taxa whose functions are relatively unknown, despite their potential importance for host-microbe interactions and nutrient cycling in kelp forest ecosystems. We reconstructed bacterial genomes from metagenomic samples collected from blades of the bull kelp, *Nereocystis luetkeana*, allowing us to determine the functional gene content of specific members of the kelp microbiome. These bacterial genomes spanned 6 phyla and 19 families and included common alga-associated microbial symbionts such as *Granulosicoccus*. Key functions encoded in kelp-associated bacterial genomes included dissolved organic matter assimilation, alginate metabolism, vitamin B_12_ biosynthesis, and nitrogen reduction from nitrate and urea to ammonium, potentially providing the host kelp with vitamins and reduced nitrogen.

## INTRODUCTION

Associations between eukaryotic hosts and microbial communities are ubiquitous, yet we are just beginning to discover the functions of the microbial partners in many of these associations. Kelp, brown macroalgae in the order *Laminariales*, are among the fastest-growing and most productive marine algae (1, 2). Canopy-forming kelp species create structural habitat in temperate and arctic coastal regions worldwide (3). Photosynthetic kelp blades are covered by a dense and diverse microbiome, with up to 10^7^ bacterial cells per cm^2^ of kelp tissue (4, 5). Microbes associated with large habitat-forming organisms can directly influence ecosystem-level biogeochemical cycles (6), yet we know little about the functional role of the kelp microbiome.

Microbial communities associated with macroalgae are often specific to each host species and distinct from microbial communities in the surrounding seawater (7–9), yet certain bacterial groups are also shared among diverse host macroalgae (10, 11). For example, *Granulosicoccus* is an abundant bacterial symbiont on many kelps (9, 12–15), the brown alga *Fucus* (16, 17), and other diverse macroalgal hosts (18). The ubiquity of this genus points to a potentially important role, but the metabolic functions of this pervasive macroalgal symbiont are unknown.

Microbial metabolisms can greatly influence the biology of their hosts. For example, bacteria associated with phytoplankton can provide fixed or reduced nitrogen and cofactors such as vitamin B_12_ to their host algae in exchange for organic carbon (19, 20). Microbial nitrogen metabolisms such as nitrate and nitrite reduction have been identified in the microbiome of Macrocystis pyrifera (21), and nitrogen fixation was quantified from *M. pyrifera* blades (22). Further, the microbiome of *M. pyrifera* contained nitrite reductase genes from diverse bacterial groups, which may have increased ammonium availability to their host kelp under experimental nitrogen limitation (23). In addition to transforming nitrogen, bacteria may metabolize kelp-derived carbon. Kelp release ~16% of carbon fixed through photosynthesis into the surrounding seawater as dissolved organic carbon (DOC) (24, 25). Bacteria in the surrounding seawater consume kelp-derived DOC (26, 27), and cultured bacterial isolates from the kelp surface degrade polysaccharides such as alginate, fucoidan, laminarin, and mannitol (28, 29). However, we know little about the metabolic capabilities of kelp-associated bacterial groups in nature.

Here, we used a genome-resolved metagenomics approach to determine the functional roles and metabolic capabilities of bacterial symbionts associated with photosynthetic blades of the canopy-forming bull kelp (Nereocystis luetkeana). The bull kelp microbiome is comprised of a few microbial taxa that persist across geographic locations (9), colonize new tissues rapidly (30), reach high cell densities, and display repeatable micrometer-scale spatial structure (5). We reconstructed 79 bacterial metagenome-assembled genomes (MAGs) from bull kelp blade tissues, spanning 6 bacterial phyla and 19 families. By assembling bacterial genomes from kelp blades over 3 consecutive years, we tested whether the annual life history of *N. luetkeana* affects the continuity of bacterial taxa across years. We evaluated whether microbial metabolisms that are likely to interact with host kelp metabolisms or contribute to nutrient cycling in kelp forest ecosystems are present in kelp-associated bacterial genomes by searching for genes related to dissolved organic matter (DOM) assimilation, laminarin and alginate metabolism, nitrogen metabolism, and vitamin B_12_ biosynthesis. We tested the hypothesis that kelp-associated microbes have the capacity to consume dissolved organic matter (DOM) by searching for cell membrane DOM transport proteins in the assembled bacterial genomes, which are used by bacteria to assimilate DOM (31, 32). Finally, we determined the metabolic functions of the most abundant member of the kelp microbiome, *Granulosicoccus*. Pangenomic comparisons of kelp-associated *Granulosicoccus* MAGs to other available *Granulosicoccus* genomes facilitated the discovery of novel functions associated with this bacterial genus.

## RESULTS

### Bacterial genomes assembled from kelp blade surface swabs and whole kelp tissues.

Shotgun metagenomic sequencing of 7 samples collected from blades of *N. luetkeana* resulted in 156.4 million high-quality short reads, with an average of 22 million reads per sample (see Table S1 in the supplemental material). We manually reconstructed 79 MAGs from both whole kelp blade tissue samples and blade surface swabs. These MAGs belong to 6 different bacterial phyla, including 16 from the class *Gammaproteobacteria*, 15 from the class *Alphaproteobacteria*, 22 of *Bacteroidetes*, 13 of *Verrucomicrobia*, 9 of *Bdellovibrionota*, 2 of *Planctomycetes*, and 2 of *Patescibacteria* (Table S2 and Fig. S1). Of the 79 MAGs, 13 were redundant (>99% average nucleotide identity [ANI]), yielding a final data set of 66 unique or nonredundant MAGs (Table S2). Metagenomic assemblies from whole kelp tissue samples contained 39.0 to 49.8% kelp host DNA, so despite the mix of kelp and bacterial genomes, approximately half of the sequences were bacterial. Read recruitment of short reads from each metagenome sample to the assembled MAGs revealed that surface swabs were more effective at capturing bacterial genomes, with less host contamination. Metagenomic samples assembled from kelp surface swabs yielded a higher percentage of sequences that mapped to bacterial genomes (88.2%) than did samples assembled from kelp tissues (37.3%; Table S1). This also indicates that the MAGs captured most of the bacterial reads present in metagenomic samples.

10.1128/msystems.01422-21.1 FIG S1 A maximum-likelihood phylogenetic tree showing the 66 nonredundant bacterial MAGs assembled from kelp blades, with branches colored by bacterial phyla. Bootstrap support values are indicated by different-colored circles at the nodes. Download FIG S1, TIF file, 1.4 MB. Copyright © 2022 Weigel et al. 2022 Weigel et al. https://creativecommons.org/licenses/by/4.0/ This content is distributed under the terms of the Creative Commons Attribution 4.0 International license.

10.1128/msystems.01422-21.4 TABLE S1 List of metagenome samples, collection information, and quality control information about metagenomic sequence reads. Download Table S1, XLSX file, 0.01 MB. Copyright © 2022 Weigel et al. 2022 Weigel et al. https://creativecommons.org/licenses/by/4.0/ This content is distributed under the terms of the Creative Commons Attribution 4.0 International license.

10.1128/msystems.01422-21.5 TABLE S2 List of all 79 metagenome-assembled genomes (MAGs), including unique and redundant MAGs, and associated information such as genome length, GC content, completion, redundancy, and taxonomic classification. Download Table S2, XLSX file, 0.02 MB. Copyright © 2022 Weigel et al. 2022 Weigel et al. https://creativecommons.org/licenses/by/4.0/ This content is distributed under the terms of the Creative Commons Attribution 4.0 International license.

### Detection and abundance of bacterial genomes that persist over multiple years.

**FIG 1:**
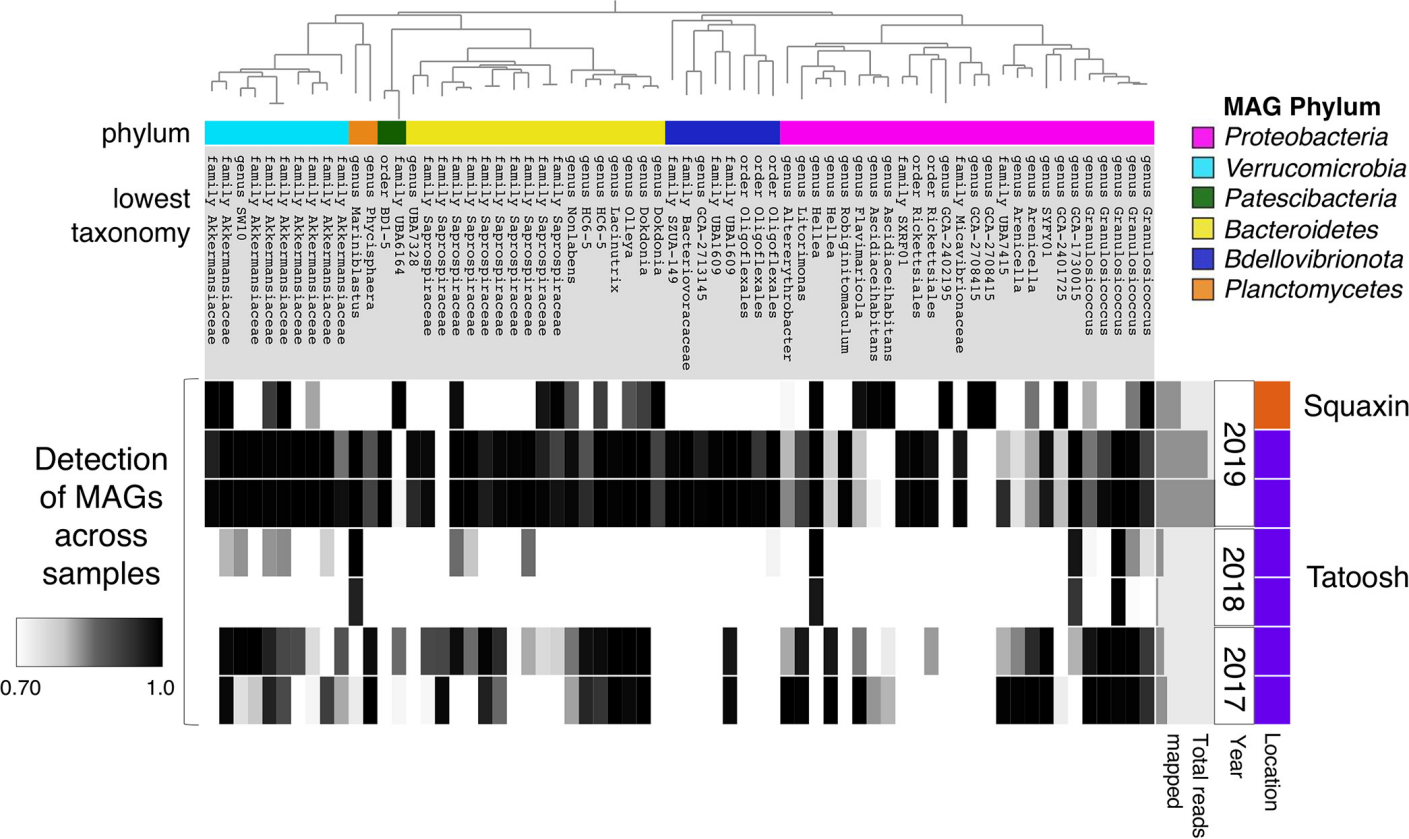
A maximum-likelihood phylogenetic tree of the 66 unique metagenome-assembled genomes (MAGs) (top), colored by bacterial phylum with the lowest taxonomy listed for each MAG, showing the detection of MAGs (columns) across different kelp metagenome samples (rows). MAGs are considered present in a sample if at least 70% of the genome is covered by at least one short read from that sample, so detection ranges from 0.70 to 1.0 (black and gray bars in the heatmap). White bars in the heatmap indicate the absence of a MAG in that sample, where less than 70% of the genome is covered by short reads from that sample. Samples are grouped by location and year, and the small bar graph to the right shows the total number of short reads mapped to all MAGs in each metagenome sample.

We assembled bacterial genomes collected from *N. luetkeana* blades on Tatoosh Island in 2017, 2018, and 2019 and from Squaxin Island in 2019. Read recruitment of short reads from each metagenome to assembled bacterial genomes revealed that 31 MAGs (47% of the total number of unique MAGs) were detected (defined as >70% of the genome covered by short reads from that sample) across 2 or more years, and 15 were detected in both locations (Fig. 1). In contrast, 30 MAGs (45%) were detected in only a single year or at a single location. A single MAG of *Granulosicoccus* (g4_MAG_00004) was detected in all samples from Tatoosh Island in 2017, 2018, and 2019 (Fig. 1). However, metagenomes from 2018 had a low rate of detection of bacterial MAGs (Fig. 1), likely because they were assembled from whole kelp tissues. Excluding the 2018 samples, 26 MAGs (39%) were detected in both 2017 and 2019 on Tatoosh Island.

**FIG 2:**
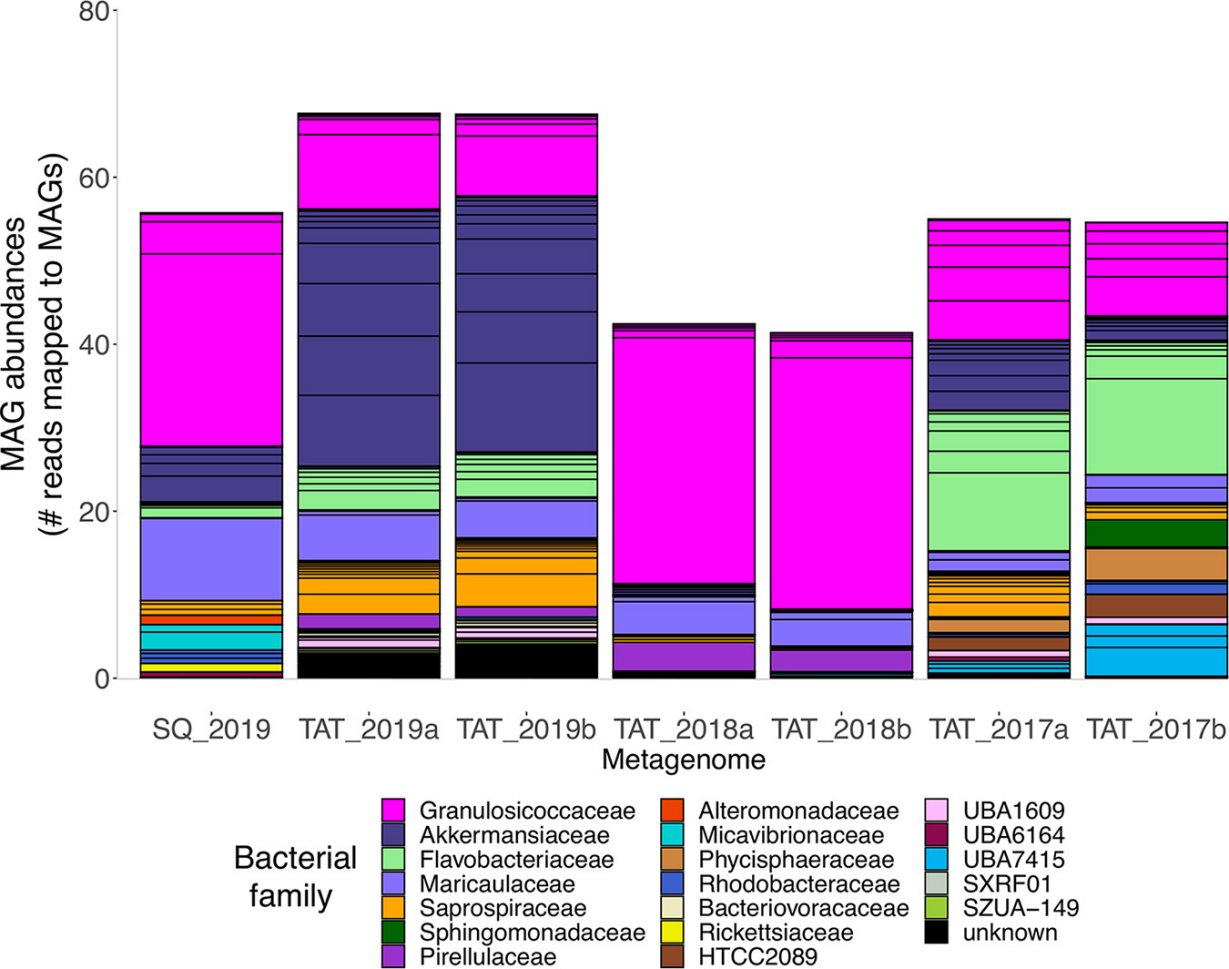
Abundance of kelp-associated MAGs, colored by bacterial family, within each metagenome sample. Black outlines within each colored bar indicate distinct MAGs belonging to each family. On the *x* axis, metagenomes from Tatoosh (TAT) and Squaxin (SQ) are indicated along with sampling year. Abundance is defined as the mean coverage of a MAG (number of metagenomic short reads mapped across the genome) divided by that sample’s overall mean coverage. For example, an abundance value of 10 indicates that the MAG’s mean coverage is 10 times that of the mean for all MAGs in that sample. See Table S3 in the supplemental material for abundance values of individual MAGs in metagenome samples.

While detection indicates the presence or absence of a MAG, abundance reveals the relative proportions of MAGs within a sample, based on the number of metagenomic short reads that mapped to each MAG. *Granulosicoccus* was the most abundant bacterial genome assembled from kelp at both locations (Fig. 2). Together, the 5 nonredundant MAGs of *Granulosicoccus* recruited an average of 40% of the total metagenomic reads across samples, with a range of 12% to 75% per sample. Different genomes of *Granulosicoccus* were differentially abundant at each location (Table S3), which are likely distinct species based on their ANI of 82% (Fig. S2). The next most abundant bacterial genomes included multiple MAGs from the family *Akkermansiaceae* (phylum *Verrucomicrobia*), *Dokdonia* (family *Flavobacteriaceae*), and *Hellea* (family *Maricaulaceae*) (Fig. 2). Together, just 8 MAGs including 4 of *Granulosicoccus*, 2 of *Akkermansiaceae*, 1 *Dokdonia*, and 1 *Hellea* accounted for 41% to 82% of all short reads mapped to kelp metagenomes (Table S3). Other abundant taxa included MAGs from the family *Saprospiraceae* (phylum *Bacteroidetes*), *Mariniblastus* (family *Pirellulaceae*), and *Lacinutrix* (family *Flavobacteriaceae*) (Fig. 2; Table S3).

10.1128/msystems.01422-21.2 FIG S2 Phylogeny of *Granulosicoccus* genomes, including 8 kelp-associated MAGs and two reference genomes, Granulosicoccus antarcticus and *Granulosicoccus* MAG 002746645. For each clade, the minimum average nucleotide identity (ANI) among all genomes is listed. The kelp-associated *Granulosicoccus* MAGs from this study fall into four distinct clades with >98% ANI, indicated by pink circles, likely representing four distinct species. Download FIG S2, TIF file, 0.4 MB. Copyright © 2022 Weigel et al. 2022 Weigel et al. https://creativecommons.org/licenses/by/4.0/ This content is distributed under the terms of the Creative Commons Attribution 4.0 International license.

10.1128/msystems.01422-21.6 TABLE S3 Abundance of MAGs across kelp metagenome samples. Abundance is defined as the mean coverage of a MAG (number of metagenomic short reads mapped across the genome) divided by that sample’s overall mean coverage. The asterisks indicate the 8 most abundant MAGs, which together accounted for 56 to 96% of all short reads mapped to kelp metagenomes. Download Table S3, XLSX file, 0.02 MB. Copyright © 2022 Weigel et al. 2022 Weigel et al. https://creativecommons.org/licenses/by/4.0/ This content is distributed under the terms of the Creative Commons Attribution 4.0 International license.

### Widespread occurrence of genes for DOM transport by diverse bacteria.

**FIG 3:**
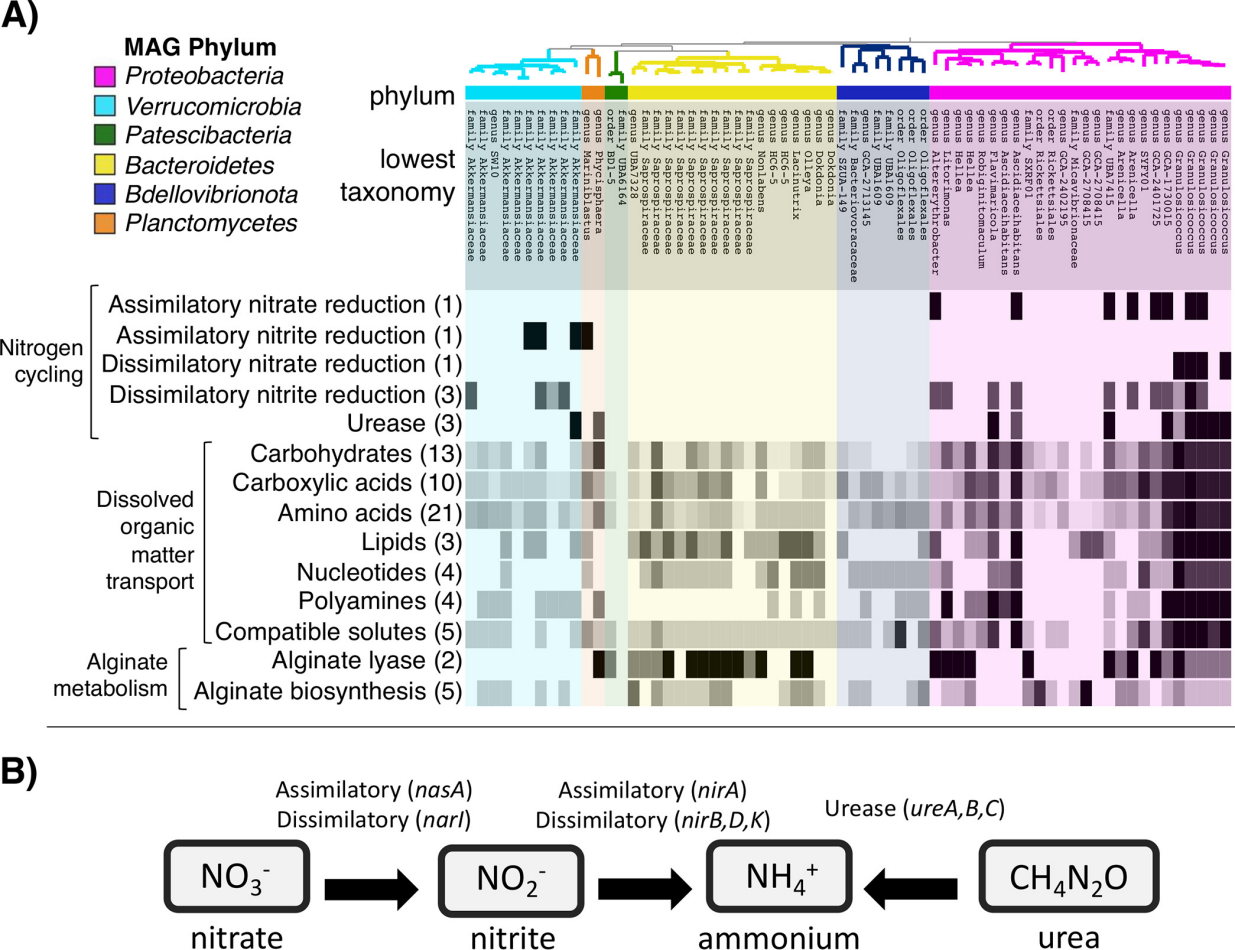
(A) Presence and absence of genes (rows) involved in nitrogen cycling, dissolved organic matter transport, and alginate metabolism across kelp-associated bacterial genomes (columns). The tips of the phylogeny represent the 66 unique bacterial genomes (MAGs), colored by phylum, with the lowest taxonomic level and name. The number of genes in each category is listed in parentheses to the left of the heatmap, and the shade of the heatmap indicates the number of genes present in each MAG. Dissolved organic matter transporter genes are grouped by substrate type. See Tables S4 and S5 in the supplemental material for expanded lists of DOM transport, nitrogen cycling, and alginate metabolism gene presence or absence across MAGs. (B) Diagram of the nitrogen transformation genes contained in kelp-associated bacterial genomes listed above.

We detected the presence of 72 genes for dissolved organic matter (DOM) assimilation among the 66 unique bacterial genomes assembled from the kelp surface (Fig. 3A; Table S4). Genes identified in kelp-associated bacterial genomes transported diverse DOM substrates, including amino acids, oligopeptides, polyamines, lipids (long-chain fatty acids and glycerol), nucleotides, carbohydrate sugars, carboxylic acids, and solutes (Fig. 3A; Table S4). DOM transport proteins were present in members of all bacterial phyla and in almost every bacterial genome, with the exception of one MAG from the phylum *Patescibacteria* and family UBA6164 (Fig. 3A). Most bacterial genomes contained multiple genes for DOM transport, with a median of 14 genes per genome (Table S4). Genomes of *Granulosicoccus* contained the highest number of DOM transporters, with a range of 53 to 59 distinct transport protein genes per genome (Fig. 3A; Table S4). Many of these genes are ATP-binding cassette (ABC)-type transporters, fueled by ATP hydrolysis to actively translocate substrates across the bacterial cell membrane (33). ABC-type transporters involved in transporting amino acids and oligopeptides and sugars such as xylose, ribose, and arabinose were present in diverse bacterial genomes (Table S4). In addition to ABC-type transporters, bacterial genomes contained genes for permeases that facilitate transport across the membrane (e.g., fucose permease), as well as tripartite ATP-independent periplasmic (TRAP)-type transporters for mannitol and carboxylic acids (Table S4).

10.1128/msystems.01422-21.7 TABLE S4 Expanded list of dissolved organic matter (DOM) transporter genes from Fig. 3A, grouped by substrate type, with COG database numbers and descriptions, including the presence (Y) or absence () of each gene in each MAG. Download Table S4, XLSX file, 0.03 MB. Copyright © 2022 Weigel et al. 2022 Weigel et al. https://creativecommons.org/licenses/by/4.0/ This content is distributed under the terms of the Creative Commons Attribution 4.0 International license.

### Alginate degradation, and production, by kelp surface bacteria.

Alginate lyase genes, including poly(beta-d-mannuronate) lyase (*algL*) and oligoalginate lyase (*alg17C*), were present in 4 bacterial phyla and 10 bacterial families but were most common in MAGs from the *Bacteroidetes* (family *Saprospiraceae*), *Alphaproteobacteria* (*Hellea*, *Litorimonas*, and *Altererythrobacter*), and *Gammaproteobacteria* (*Arenicella*, families UBA7415 and *Granulosicoccaceae*) (Fig. 3A; Table S5). While some bacteria degrade alginate, others synthesize this polysaccharide as a component of extracellular biofilm formation. One *Alphaproteobacteria* member in the genus GCA-2708415 (family *Micavibrionaceae*) contained 5 alginate biosynthesis genes (Fig. 3A; Table S5). The *algE* gene, responsible for the export of synthesized alginate (34), was present in *Granulosicoccus* and the *Alphaproteobacteria* member GCA-2708415. In contrast to alginate lyase genes, genes encoding laminarinase enzymes, also known as endo-1,3-beta-d-glucosidases, were surprisingly absent from kelp-associated bacterial genomes (Table S5). Additional carbohydrate metabolism genes, likely to play a role in degradation of mucin and sulfated polysaccharides, were abundant in the genome of *Akkermansiaceae*, including 66 sulfatase genes, 3 ABC-type polysaccharide transporter genes, and 24 *N*-acetylglucosamine metabolism genes.

10.1128/msystems.01422-21.8 TABLE S5 Expanded list of nitrogen cycling, laminarin, and alginate metabolism genes from Fig. 3A, with KEGG database numbers and descriptions, including the presence (Y) or absence () of each gene in each MAG. Download Table S5, XLSX file, 0.04 MB. Copyright © 2022 Weigel et al. 2022 Weigel et al. https://creativecommons.org/licenses/by/4.0/ This content is distributed under the terms of the Creative Commons Attribution 4.0 International license.

### Nitrogen metabolisms in the kelp microbiome.

Kelp-associated bacterial genomes from the *Proteobacteria*, *Verrucomicrobia*, and *Planctomycetes* contained genes for dissimilatory nitrate and nitrite reduction and urea hydrolysis (Fig. 3A and B). MAGs in the family *Akkermansiaceae* (phylum *Verrucomicrobia*) contained genes for dissimilatory nitrite reduction (*nirA*, *nirB*, and *nirD*; Table S5). One *Akkermansiaceae* member also had the ability to hydrolyze urea with genes encoding the three urease subunits (*ureABC*). The *Planctomycetes Mariniblastus* contained a gene for nitrite reduction (*nirA*), and *Phycisphaera* had *ureAB* and *ureC*. The greatest diversity of nitrogen metabolisms was found within the *Proteobacteria* (Fig. 3A; Table S5). Multiple MAGs contained both nitrate and nitrite reduction genes, indicating the potential for reduction from nitrate to ammonium, including *Altererythrobacter*, *Ascidiaceihabitans*, *Arenicella*, and *Granulosicoccus* (Fig. 3A; Table S5). *Proteobacteria* with urease genes (*ureABC*) included *Ascidiaceihabitans*, *Flavimaricola*, *Arenicella*, and *Granulosicoccus* (Fig. 3A; Table S5). *Granulosicoccus* MAGs contained genes for assimilatory and dissimilatory nitrate reduction (*nasA* and *narI*, respectively), dissimilatory nitrite reduction (*nirBDK*), and urease (Fig. 3A and B).

### *Granulosicoccus* pangenome reveals high genomic diversity and diverse metabolisms.

We assembled 8 *Granulosicoccus* genomes, with an average genome length of 4,292,108 bp and an average GC content of 49.02%. *Granulosicoccus* MAGs were assembled from samples collected in all 3 years and at both locations and range in completion from 77.5 to 98.6% complete, with 6 MAGs >90% complete with <5% contamination (Table S2). The demarcation for a bacterial species using whole-genome average nucleotide identity (ANI) is typically ≥95% (35), while ANI values across genera average 73% (36). The mean ANI between *Granulosicoccus* MAGs in this study was 81.3%, but the MAGs clustered into 4 distinct clades with an ANI of >98% within each clade, likely representing 4 distinct species within the genus *Granulosicoccus* (Fig. S2). We analyzed the pangenome of these 8 *Granulosicoccus* genomes together with genomes of Granulosicoccus antarcticus type strain IMCC3135 (37) and *Granulosicoccus* MAG 002746645 (38). The mean ANI between MAGs in this study and the reference genomes was 71.9% for *G*. *antarcticus* and 70.2% for *Granulosicoccus* MAG 002746645. Two kelp-associated MAGs were more closely related to *G*. *antarcticus* (>72% ANI) than the others (Fig. S2).

The *Granulosicoccus* pangenome contained a core genome of 6,222 genes shared among all 10 genomes (15% of the total number of genes in the pangenome), a large accessory genome of 26,873 genes that were present in at least two but not all genomes (66% of the pangenome), and 7,684 unique genes (19%) that were present in only a single genome (Fig. S3). Genes related to amino acid, carbohydrate, and lipid transport and metabolism were among the most abundant gene clusters in the core genome (Table S6), along with essential cellular functions such as transcription, translation, and cell wall biogenesis. The core genome also contained many gene clusters related to cell motility (Table S6).

10.1128/msystems.01422-21.3 FIG S3 Pangenome representing 10 *Granulosicoccus* genomes, including the 8 kelp-associated MAGs and two reference genomes, Granulosicoccus antarcticus type strain IMCC3135 and *Granulosicoccus* MAG 002746645. Each concentric circle represents a bacterial genome, broken down into core gene clusters shared by all 10 genomes, accessory gene clusters present in at least 2 but not all genomes, and unique gene clusters present in only a single genome. Black indicates the presence of a gene cluster in a genome. The inset bar graphs show genome completion in blue, redundancy in red, GC content in green, and genome length in gray. Download FIG S3, TIF file, 1.1 MB. Copyright © 2022 Weigel et al. 2022 Weigel et al. https://creativecommons.org/licenses/by/4.0/ This content is distributed under the terms of the Creative Commons Attribution 4.0 International license.

10.1128/msystems.01422-21.9 TABLE S6 List of core gene clusters in the *Granulosicoccus* pangenome. Download Table S6, XLSX file, 0.01 MB. Copyright © 2022 Weigel et al. 2022 Weigel et al. https://creativecommons.org/licenses/by/4.0/ This content is distributed under the terms of the Creative Commons Attribution 4.0 International license.

**FIG 4:**
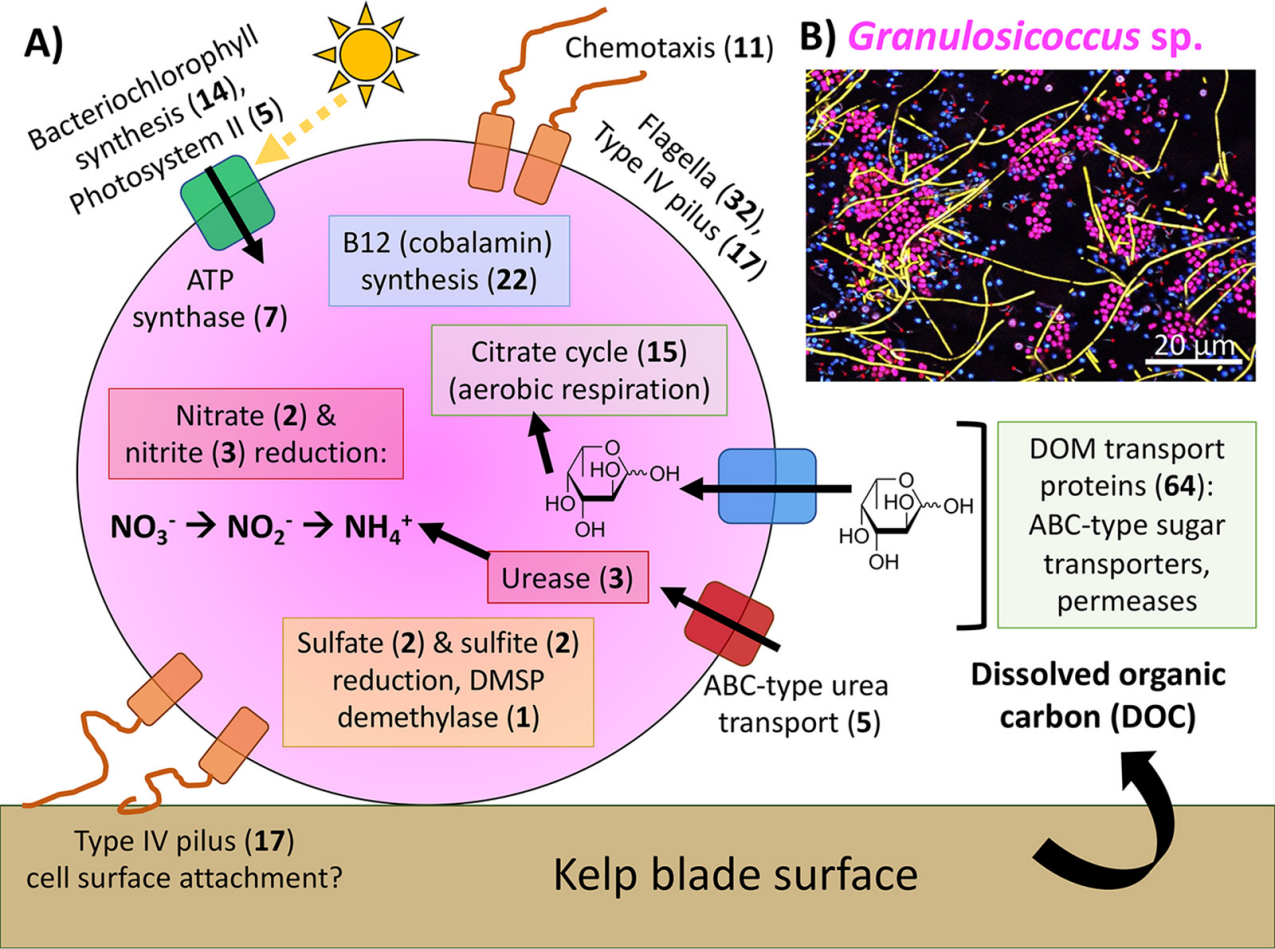
(A) Conceptual model of the functions and metabolisms of *Granulosicoccus* that are important to its role as a macroalgal symbiont, based on functional gene annotations of 8 MAGs assembled from bull kelp blades. Bold numbers in parentheses indicate the number of unique genes associated with each function. (B) Image of the micrometer-scale spatial structure of the *N. luetkeana* surface microbiome depicting clusters of magenta *Granulosicoccus* cells (coccus shaped), adapted with permission from the work of Ramírez-Puebla et al. (5).

*Granulosicoccus* MAGs in this study contained diverse metabolic genes related to DOM transport, nitrogen and sulfur transformation, motility and chemotaxis, aerobic respiration, and cobalamin (B_12_) synthesis (Table 1 and Fig. 4). *Granulosicoccus* contained 15 genes for aerobic respiration via the citrate cycle (Fig. 4). Surprisingly, *Granulosicoccus* genomes assembled from the kelp surface contained genes for bacteriochlorophyll synthesis and photosystem II (PSII) reaction center proteins (Table 1), making them a novel clade of aerobic anoxygenic phototrophic (AAP) bacteria (detailed below). *Granulosicoccus* MAGs contained 64 different genes encoding DOM transport proteins (Fig. 4; Table S4). Similar to previously reported motility genes in *G. antarcticus* (37), all 8 MAGs contained genes for synthesizing flagella (32 genes) and type IV pili (17 genes), and they contained 11 genes related to chemotaxis (Fig. 4, Table 1, and Table S7).

**TABLE 1:** Functional categories and metabolisms present in the *Granulosicoccus* pangenome^*a*^

Functional category	*G. antarcticus*	*Granulosicoccus* MAG 002746645	*Granulosicoccus* MAGs (this study)
Synthesis of bacteriochlorophyll			
Magnesium chelatase (*bchHI*)	−	−	7/8
Bacteriochlorophyllide reductase (*bchXYZ*)	−	−	7/8
Light-independent protochlorophyllide reductase (*chlLNB*)	−	−	7/8
Photosystem II			
Light-harvesting complex 1 alpha chain (*pufAB*)	−	−	7/8
Photosystem II reaction center (*pufLM*)	−	−	7/8
Photosynthetic reaction center cytochrome *c* subunit	−	−	7/8
Nitrogen metabolisms			
Nitrate reduction (NO_3_ to NO_2_)	+	−	4/8
Nitrite reduction (NO_2_ to NH_4_)	+	−	6/8
Urease (CH_4_N_2_O to NH_4_ and CO_2_)	+	−	8/8
Urea transporter	+	−	6/8
Sulfur metabolisms			
Assimilatory sulfate reduction (sulfate to hydrogen sulfide)	+	−	8/8
Thiosulfate oxidation by sox (thiosulfate to sulfate)	+	−	5/8
Sulfide oxidation	+	+	0/8
DMSP transformation	+	−	6/8
Vitamin B_12_ (cobalamin) biosynthesis			
Corrin ring biosynthesis	+	+	1/8
Cobalt insertion into corrin ring (anaerobic pathway)	−	−	1/8
Cobalt insertion into corrin ring (aerobic pathway)	+	+	8/8
Final B_12_ biosynthesis and repair	+	+	7/8
Catalyzes B_12_ into coenzyme form	+	+	8/8
B_12_ membrane transporter	+	+	7/8
Motility and chemotaxis			
Motility—flagella	+	+	8/8
Motility—type IV pilus	+	+	8/8
Chemotaxis	+	+	8/8

a For the genomes of *G. antarcticus* and *Granulosicoccus* MAG 002746645, presence (+) or absence (−) of each function is indicated. For the kelp-associated *Granulosicoccus* MAGs, *X*/8 indicates the number of genomes containing genes for each function out of the 8 assembled genomes.

10.1128/msystems.01422-21.10 TABLE S7 Detailed list of functional gene presence (Y) or absence () across all 10 *Granulosicoccus* genomes, with KEGG database numbers and descriptions. Download Table S7, XLSX file, 0.02 MB. Copyright © 2022 Weigel et al. 2022 Weigel et al. https://creativecommons.org/licenses/by/4.0/ This content is distributed under the terms of the Creative Commons Attribution 4.0 International license.

*Granulosicoccus* MAGs contained genes to transform both nitrogen and sulfur. Genomes of *Granulosicoccus* contained dissimilatory nitrate (*narI*) and nitrite (*nirB*, *nirD*, and *nirK*) reduction genes, indicating the potential for complete dissimilatory nitrate reduction to ammonium (Fig. 3A and Table 1). *G. antarcticus* contained additional nitrate reductases (*narG*, *narY*, and *narI*) that were not present in the kelp-associated MAGs (Table S7). *G. antarcticus* and kelp-associated *Granulosicoccus* MAGs contained urease genes (*ureABC*) and urea transport proteins (Fig. 3A and Table 1). *Granulosicoccus* genomes contained sulfur metabolism genes including assimilatory sulfate reduction (*cysNC*, *cysH*, *cysI*, and *cysJ*) and *sox* genes for thiosulfate oxidation to sulfate (Table 1). Closely related bacteria in the family *Granulosicoccaceae* are capable of chemolithotrophic growth by oxidizing sulfur compounds (39), but the function of these sulfur metabolisms in kelp-associated *Granulosicoccus* has yet to be determined (37). As reported previously for *G. antarcticus* (37), kelp-associated MAGs of *Granulosicoccus* contained dimethylsulfoniopropionate (DMSP) demethylase (*dmdA*), the only enzyme known to demethylate DMSP (37), an organic sulfur compound produced by algae that plays a significant role in the global sulfur cycle (40).

Finally, *Granulosicoccus* MAGs contained many of the genes necessary for cobalamin (vitamin B_12_) synthesis (Fig. 4; Table S7), a vitamin generally lacking in host macroalgae (41). While only one MAG (g3_MAG_00002) contained all 22 genes necessary for complete synthesis of B_12_, including 11 genes involved in corrin ring synthesis, 7 out of 8 MAGs contained at least 10 genes for B_12_ biosynthesis (Table 1; Table S7). Further, all 8 MAGs contained the genes to catalyze B_12_ into its coenzyme form as well as insert cobalt into the corrin ring through the aerobic pathway (*cobS* and *cobT*), and 7 out of 8 genomes contained the B_12_ membrane transporter gene *btuB* (Table 1; Table S7). Interestingly, the MAG with a complete biosynthesis pathway contained genes for insertion of cobalt into the corrin ring through both aerobic and anerobic pathways (Table S7). Other bacterial taxa contained many of the required genes for B_12_ biosynthesis, including the *Gammaproteobacteria* UBA7415 (16 genes) and *Arenicella* (9 genes), and the *Alphaproteobacteria Ascidiaceihabitans* (11 genes) and *Flavimaricola* (14 genes).

While a full kelp genome would confirm that *N. luetkeana* requires B_12_, we found genes for the B_12_-dependent (cobalamin-binding) methylmalonyl coenzyme A (CoA) mutase (MCM) in the partial host kelp genomes in both samples extracted from whole kelp tissues. If the host kelp has this B_12_-dependent enzyme, it requires vitamin B_12_ (42, 43) and may be dependent on associated bacteria for B_12_ synthesis. Using a protein BLAST search, these MCM amino acid sequences from *N. luetkeana* matched with 93% sequence identity to an unknown protein from the brown alga Ectocarpus siliculosus and with >80% sequence identity to B_12_-dependent methylmalonyl-CoA mutase proteins from other closely related eukaryotes, including Phytophthora parasitica and Phytophthora cinnamomi (eukaryotes in the *Oomycota*, closely related to kelp), indicating that these are eukaryotic MCM genes.

### *Granulosicoccus* as a new lineage of aerobic anoxygenic phototrophic bacteria.

**FIG 5:**
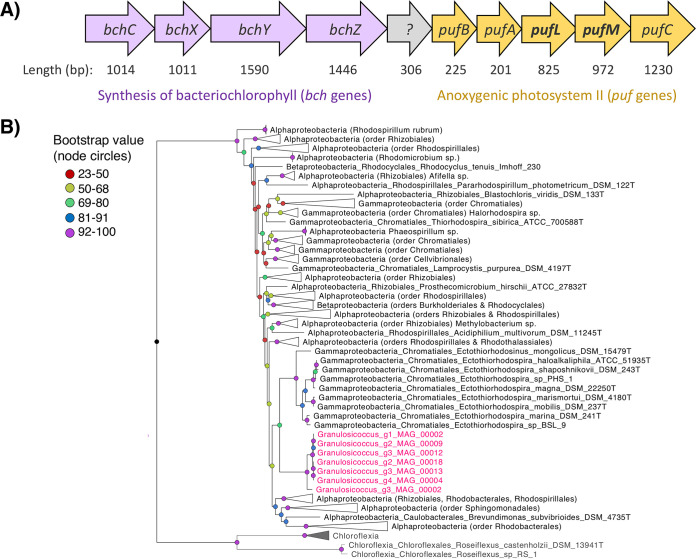
(A) Gene cluster containing 9 sequential genes for the synthesis of bacteriochlorophyll (*bch*) and anoxygenic photosystem II proteins (*puf*) from the genome of *Granulosicoccus* g4_MAG_00004 (contig 000000000044_split_00005). Photosystem II proteins include the light-harvesting complex 1 alpha and beta chains (*pufAB*) and the photosystem II reaction center subunits (*pufLMC*). Genes are represented by arrows, and gene lengths in nucleotide base pairs are listed below each arrow. (B) Maximum-likelihood phylogenetic tree showing the position of *Granulosicoccus pufLM* sequences (indicated in pink) in relation to photosystem II reaction center *pufLM* sequences from other known lineages of aerobic anoxygenic phototrophic bacteria. Bootstrap support values are color coded at the nodes. The clade containing sequences from *Granulosicoccus* and *Ectothiorhodospira* has a bootstrap support of 76.

Seven out of eight of the *Granulosicoccus* MAGs in our study contained a full suite of genes that typify the aerobic anoxygenic phototrophic bacteria (44), including genes for bacteriochlorophyll synthesis and photosystem II reaction center proteins (Fig. 5A and Table 1). There were 14 genes for bacteriochlorophyll synthesis, including magnesium chelatase (*bchHI*), bacteriochlorophyllide reductase (*bchXYZ*), and protochlorophyllide reductase (*chlLNB*) genes, and 5 genes for harvesting light energy through photosystem II reaction center proteins (*pufABC* and *pufLM*; Table 1 and Table S7). Within *Granulosicoccus* genomes, gene clusters contained 9 sequential genes encoding bacteriochlorophyll synthesis and photosystem II reaction center proteins (Fig. 5A). Surprisingly, neither *G. antarcticus* IMCC3135 nor *Granulosicoccus* MAG 002746645 had these genes, except for a single magnesium chelatase gene (*bchI*) in *G. antarcticus*, which lacks the other bacteriochlorophyll synthesis genes.

To confirm that kelp-associated *Granulosicoccus* are a novel lineage of aerobic anoxygenic phototrophic bacteria, we inferred a phylogenetic tree with the photosystem II reaction center genes (*pufL* and *pufM*) from each *Granulosicoccus* MAG together with a reference database of 167 *pufL* and *pufM* sequences from reference 44. Photosystem II protein sequences from *Granulosicoccus* MAGs in this study form a highly supported clade (bootstrap support 76) with *pufLM* sequences from *Ectothiorhodospira*, which also belong to the order *Chromatiales* (Fig. 5B). However, we note that *pufM* protein sequences within the *Proteobacteria* are closely related, and sequences from the *Alpha*-, *Beta*-, and *Gammaproteobacteria* do not form monophyletic clades (Fig. 5B).

Finally, *Granulosicoccus* does not appear to have the enzymes necessary to fix carbon with this light energy. Key enzymes for the reverse tricarboxylic acid cycle (45) were absent, including ATP citrate lyase (*aclA* and *aclB* genes) and the CO_2_-fixing enzyme pyruvate ferredoxin oxidoreductase (*porABCD*). Further, *Granulosicoccus* lacks genes that encode the CO_2_-fixing enzyme of the Calvin cycle, RuBisCo (*cbbLM* and *rbcLS*). Therefore, the kelp-associated *Granulosicoccus* MAGs in this study are most likely photoheterotrophs, harvesting light energy with bacteriochlorophyll and photosystem II as an extra energy source while consuming organic carbon, likely from kelp-derived DOC (Fig. 4).

## DISCUSSION

### Bacterial taxa persist over multiple years and distant geographic locations.

*N. luetkeana* is an annual kelp species that grows from microscopic gametophytes into large sporophytes (5 to 30 m tall) by midsummer (46). Adult sporophytes generally do not survive through the winter (47), especially at very wave-exposed sites like Tatoosh Island. Despite this life history, 31 MAGs (47% of the total) were detected across multiple years on *N. luetkeana* blades from Tatoosh. This remarkable continuity demonstrates that similar bacterial genomes can persist across years on a host that is only seasonally abundant, suggesting the presence of a bacterial reservoir during the months when bull kelp sporophytes are absent. Potential reservoirs include the seawater, where kelp-associated bacteria are found at low abundances (9), rocky substrates (15), perennial kelp species (11), or overwintering kelp gametophytes, which deserve further study. Because many of the abundant MAGs and those that persisted across years contained genes for nitrogen reduction and B_12_ biosynthesis, discussed below, these abundant and persistent members of the kelp microbiome may be functionally important to the host kelp.

MAGs persisted spatially as well as temporally, with 15 MAGs (23% of the total) detected on bull kelp from Tatoosh and Squaxin populations, which are separated by the length of Puget Sound and the Strait of Juan de Fuca (~300 km). Kelp from Squaxin Island have lower bacterial cell abundances (5) and a different composition of microbes than do kelp from Tatoosh Island (9). Here, we found that *Granulosicoccus* was the most abundant MAG at both sites in 2019. However, two different genomes of *Granulosicoccus* that are likely to be distinct species were differentially abundant at each location, revealing geographic differences in species-level dynamics that are likely to be missed by 16S gene sequencing. The functional gene content was nearly identical between these two differentially abundant sequence variants of *Granulosicoccus*, indicating similar functions of the dominant bacterial taxa at these geographically separated sites.

### Diverse kelp-associated bacteria have the capacity to assimilate DOM and metabolize alginate.

Heterotrophic bacteria rely on organic carbon for biomass production. Canopy-forming kelp contribute significantly to the pool of dissolved organic carbon (DOC) in seawater (48) by releasing approximately 15 to 30% of their total fixed carbon as DOC (24, 25, 27, 49). We found that nearly all members of the kelp microbiome contained genes encoding DOM membrane transport proteins, which are the primary mechanism for DOM assimilation by bacteria (31). We detected the presence of 72 diverse genes for DOM assimilation across 66 unique bacterial genomes, with a median of 14 distinct genes per genome. *Granulosicoccus* genomes contained the highest number of DOM transport genes, with a range of 53 to 59 per genome. Kelp are known to release monosaccharides including mannitol, fucose, ribose, xylose, and galactose (50, 51), and kelp-associated *Granulosicoccus* are capable of assimilating these resources (Table S4). ATP-binding cassette (ABC)-type transporters for amino acids and sugars indicate active transport into bacterial cells (33). While bacteria in the surrounding seawater also consume kelp-derived DOM (26, 27), the microbiome of the giant kelp *M. pyrifera* was enriched in transport protein genes relative to metagenomes from the surrounding seawater (21), suggesting the importance of DOM assimilation by kelp surface-associated bacteria. While we cannot infer from genomic content alone that these DOM transport proteins are being expressed, they are good indicators of potential kelp-derived DOM use by members of the kelp microbiome. Further research should use isotope tracer or culture-based studies to demonstrate that biomass production of kelp-associated bacteria is fueled by DOM released by the host kelp.

Kelp-associated bacterial genomes contained genes for alginate lyase, which degrades alginate, a brown algal cell wall polysaccharide, into oligosaccharides. Given that alginate can comprise more than 25% of kelp dry weight (52), it represents an extensive pool of organic carbon. A culture-based study from the giant kelp *Macrocystis pyrifera* reported alginate use in diverse *Gammaproteobacteria*, *Alphaproteobacteria*, and *Flavobacteriaceae* (29). Here, we found alginate lyase genes in those groups as well as the *Planctomycetes* (*Phycisphaera*) and additional families including the *Saprospiraceae*, *Granulosicoccaceae*, *Maricaulaceae*, *Sphingomonadaceae*, and UBA7415. One of the most abundant bacteria in this study, *Hellea*, was abundant on other kelps such as Laminaria setchellii (15) and Laminaria hyperborea (14). *Hellea* genomes contained many DOM assimilation and alginate lyase genes, suggesting an active role in kelp forest carbon metabolism. Surprisingly, we did not detect genes for degradation of the storage polysaccharide laminarin in kelp-associated bacterial genomes.

While some bacteria degrade alginate, others synthesize this polysaccharide as a component of biofilm or extracellular matrix formation. The pathogen Pseudomonas aeruginosa notoriously secretes alginate during biofilm formation in the lungs (34). Here, diverse kelp-associated bacteria contained genes encoding alginate biosynthesis proteins. One *Alphaproteobacteria* member (genus GCA-2708415) contained 5 alginate biosynthesis genes, including the *algE* gene responsible for alginate export across the outer membrane (34). Further research should investigate the role of alginate-producing bacteria in biofilm formation on diverse hosts, from the human gut to macroalgal surfaces.

Kelp-associated *Akkermansiaceae* MAGs contained many genes for sulfatases, which are key enzymes in the degradation of mucin (53) and other sulfated polysaccharides, including fucoidan (54, 55). *Akkermansiaceae* MAGs also contained genes for metabolizing the sugar *N*-acetylglucosamine, a component of mucin that is likely abundant in the kelp surface mucus layer. Members of the *Akkermansiaceae* are common symbionts in the healthy human gut, where they specialize in the degradation of mucin (56), and they likely play a similar role in the kelp microbiome. *Akkermansiaceae* are understudied in the environment but were abundant during spring diatom blooms, where they consumed sugars (55).

### Kelp-associated bacteria reduce oxidized nitrogen sources to ammonium.

The annual kelp *N. luetkeana* grows extraordinarily fast, producing 1 to 6 cm of new blade tissue per day (47), and rapidly assimilates dissolved inorganic nitrogen (25, 57, 58). Ammonium is the most energetically preferable form of dissolved inorganic nitrogen for algae, as it can be directly incorporated into amino acids without expending energy on intracellular nitrate reduction (59, 60). While nitrate concentrations are higher than those of ammonium on the coast of Washington during the summer (61), *N. luetkeana* blades assimilate ammonium 1.5 times faster than nitrate relative to its availability (25), indicating a preference for the more reduced form of nitrogen. Urea is a nitrogenous waste product excreted by common marine animals, including zooplankton (62), and urea can constitute a significant proportion (~20%) of the dissolved nitrogen pool in coastal seawater (63). Kelp-associated bacterial genomes from the *Proteobacteria*, *Verrucomicrobia*, and *Planctomycetes* contained one or more genes for dissimilatory nitrate and nitrite reduction and urea hydrolysis (Fig. 3B), which generate ammonium as a reduced nitrogen source that may be favorable to the host kelp.

Despite the presence of these genes, it is still unknown whether ammonium generated through dissimilatory nitrate reduction or urea hydrolysis is accessible to the host kelp. Surface-associated bacteria also compete directly with the host kelp for nitrogen in the seawater (64), and kelp-associated MAGs contained assimilatory nitrate and nitrite reduction genes. While it may seem surprising to find dissimilatory nitrate reduction, an anaerobic process, associated with a photosynthetic host, kelp blades deplete oxygen at night through respiration (65), and biofilm layers can have reduced oxygen (66). Nitrate and nitrite reduction genes were also enriched in the surface microbiome of the giant kelp *M. pyrifera* compared to seawater metagenomes (21); thus, nitrate reduction may be a common function of the kelp microbiome. Future studies should test the hypothesis that kelp-associated bacteria provide reduced nitrogen to their host kelp using ^15^N isotope tracers.

### Kelp-associated *Granulosicoccus* are motile, photoheterotrophic, nitrogen- and sulfur-transforming microbes with the potential to synthesize cobalamin (B_12_).

The genus *Granulosicoccus* (order *Chromatiales*, class *Gammaproteobacteria*) currently contains 4 species isolated from Antarctic seawater (67, 68), the seagrass Zostera marina (69), and the kelp Undaria pinnatifida (70). *Granulosicoccus* is the most abundant bacterium associated with *N. luetkeana* (9), reaching densities of up to 10^6^ cells per cm^2^ on kelp blades (5), and it associates with diverse algal hosts (18). Only a single annotated genome is available for this bacterial genus, belonging to the free-living Granulosicoccus antarcticus type strain IMCC3135 (37). Here, we reconstructed 8 MAGs of *Granulosicoccus* associated with kelp blades which share, on average, only 71.9% similarity with *G. antarcticus*. The *Granulosicoccus* genomes assembled from the kelp surface likely represent 4 new species in this genus, as they formed 4 distinct clades with ANI of >98% within each clade (Fig. S2). Kelp-associated *Granulosicoccus* had large genomes of ~4.3 Mbp (Table S2), consistent with the large genome of *G. antarcticus* that is also enriched in genes for carbohydrate and amino acid transport and metabolism relative to other bacteria in the order *Chromatiales* (37).

Kelp-associated *Granulosicoccus* are motile and likely chemotactic, providing a mechanism for colonizing kelp tissues from the seawater and reaching high abundances on new kelp blade meristem tissues (5, 12, 15, 30). *Granulosicoccus* genomes contained 32 flagellar genes and 11 chemotaxis genes, including two-component system chemotaxis proteins and genes for rotation of the flagellar motor (Table S7). The motility of *G. antarcticus* is achieved through numerous tufts of flagella (67). All *Granulosicoccus* genomes also contained up to 17 type IV pilus assembly protein genes, which are known to be involved in biofilm formation and adhesion to host cells (71). On the kelp surface, *Granulosicoccus* cells are closely associated with kelp cells at the base of the biofilm (5).

*Granulosicoccus* belongs to the order *Chromatiales*, also known as purple sulfur bacteria because they are usually phototrophic and contain carotenoid and bacteriochlorophyll pigments (44, 72). While previous isolates of *Granulosicoccus* are obligately chemoheterotrophic (37, 67), we found genomic evidence that kelp-associated *Granulosicoccus* have the capacity to harvest light energy with bacteriochlorophyll and photosystem II reaction center proteins. Genes for CO_2_ fixation were absent, suggesting a photoheterotrophic metabolism. Further, culture studies of *Granulosicoccus* show that they cannot fix carbon and require organic carbon for growth (67). PSII reaction center genes (*pufLM*) from *Granulosicoccus* MAGs formed a clade with *pufLM* genes from *Ectothiorhodospira* (Fig. 5B), another genus in the order *Chromatiales*. While many purple sulfur bacteria are anaerobic (44), *Granulosicoccus* MAGs contained genes for aerobic respiration via the citrate cycle, and *G. antarcticus* is obligately aerobic (67). Therefore, the *Granulosicoccus* from this study likely represent a novel reported lineage of aerobic anoxygenic phototrophic (AAP) bacteria. As photoheterotrophs, AAP bacteria have the capacity to generate ATP with light energy, providing extra energy for the cell that increases their growth efficiency and gives them a competitive advantage over other heterotrophic bacteria (73–75). This energetic advantage may allow them to reach high densities and form cell clusters on kelp blade surfaces (5). Canopy kelp likely provide ideal habitats for AAP bacteria that benefit from both sunlight exposure and a constant supply of organic carbon. Other notable AAP bacteria include phytoplankton-associated *Roseobacter* bacteria (73, 76), which likely consume phytoplankton-exuded DOC (75). While AAP bacteria associated with macroalgae have a constant supply of organic carbon and may not be energy limited, we hypothesize that supplemental energy from photoheterotrophy may aid *Granulosicoccus* survival during periods of non-host association.

*Granulosicoccus* MAGs contained more than 50 different genes for carbohydrate and DOM transport, likely to assimilate kelp-derived dissolved organic carbon resources (25). The most complete (98.6%) *Granulosicoccus* MAG contained 22 genes for complete biosynthesis of vitamin B_12_ (cobalamin), while 7 out of 8 MAGs contained at least 10 genes for B_12_ biosynthesis. Bacteria are the only organisms known to synthesize vitamin B_12_; thus, diverse algae rely on associated bacteria to produce vitamin B_12_ (41, 76), and brown algae are no exception (77). While a full genome of *N. luetkeana* may be necessary to confirm this finding (42), kelp host genomic content contained the B_12_-dependent enzyme methylmalonyl-CoA mutase, which requires B_12_ as a cofactor to catalyze an essential catabolic reaction (42, 43). Interactions analogous to those described here exist between phytoplankton and heterotrophic bacteria; carbohydrates and amino acids are secreted by the phytoplankton host, and associated bacteria provide reduced nitrogen and vitamins such as B_12_ (19, 20, 78).

In summary, the genomes of kelp-associated bacteria carried carbon assimilation, polysaccharide degradation, nitrogen transformation, and vitamin biosynthesis genes that may be central to kelp-microbe interactions. Kelp forests are declining globally due to numerous environmental stressors, including ocean warming (79, 80). These climatic stressors may also impact the kelp microbiome (81), heightening our need to understand the functional role of the kelp microbiome. Kelp play a critical role as habitat-forming foundation species in temperate and arctic coastal ecosystems worldwide, and we must continue to investigate how the kelp microbiome impacts kelp fitness.

## MATERIALS AND METHODS

### Sample collection, DNA extraction, and metagenomic sequencing.

Samples for metagenomic sequencing were obtained from individuals of the kelp *Nereocystis luetkeana* at a well-studied location on Tatoosh Island, WA, USA (48.39°N, 124.74°W), over three consecutive years. Samples were collected at the north-facing Main Beach site in July 2017, July 2018, and July 2019. In July 2019, additional *N. luetkeana* samples were collected from Squaxin Island in southern Puget Sound (47.18°N, 122.91°W), the southernmost location in Puget Sound where *N. luetkeana* persists. Previous research found a significantly different microbial community composition and reduced microbial cell density on kelp from Squaxin Island compared to those from Tatoosh Island (5, 9). In 2017 to 2018, whole tissue blade samples from Tatoosh Island were collected by removing 2 by 1 cm^2^ of tissue from the middle of the kelp blade with sterile scissors. In 2019, samples from both Tatoosh and Squaxin Island *N. luetkeana* populations were collected by swabbing the middle to tip of the blade for 20 s with a sterile cotton swab. All samples were immediately frozen at −20°C and transferred to −80°C for storage.

DNA was extracted from whole kelp tissues and swabs using the DNeasy Power Soil kit (Qiagen). To acquire a sufficient quantity of DNA for metagenomic library preparation (>100 ng), DNA extracts from multiple kelp samples were pooled for each individual metagenome, with the number of pooled replicates listed in Table S1 in the supplemental material. In total, 7 metagenome samples were sequenced from *N. luetkeana* blades. DNA extracts were sent to the Argonne National Laboratory for library preparation and metagenomic sequencing on an Illumina HiSeq 2500 (2 by 150 bp).

### Assembly, annotation, and binning of metagenome-assembled genomes (MAGs).

The following analyses, from sequence quality control to binning, were performed within the anvi’o 7.0 environment (82, 83). All associated code is publicly available at https://github.com/brookeweigel/Kelp_associated_bacterial_genomes. First, raw sequences from forward and reverse reads were checked for sequence quality using “filter-quality-minoche” (84). For all samples, >92% of sequence reads passed quality control, and the mean number of reads per sample after quality control was 22,339,573 (Table S1). Quality sequences were assembled into contigs using IDBA-UD (85) with a minimum contig length of 1,000. Metagenomes collected from the same kelp forest location and same year were coassembled, resulting in 4 total assemblies (Table S1). We did not assemble across multiple years or locations to avoid chimeric genomes. The command “anvi-gen-contigs-database” was used to generate contigs databases, which compute k-mer frequencies and identify open reading frames with Prodigal (86). To determine the occurrence of 22 bacterial single-copy genes in each contigs database, hidden Markov models were run using HMMER (87) with “anvi-run-hmms.” Genes in each contigs database were annotated with all 3 available databases: (i) NCBI’s Clusters of Orthologous Genes (COGs), (ii) EBI’s Pfam database, and (iii) KEGG (Kyoto Encyclopedia of Genes and Genomes). Metagenomic short reads from all 7 samples were mapped to each of the 4 coassemblies using Bowtie2 (88), and SAMtools (89) was used to produce BAM files.

To perform metagenomic binning of contigs using anvi’o, profile databases were generated from BAM files and contigs databases using “anvi-profile” with a minimum contig length of 2,500 (to visualize all contigs), and profiles for coassembled samples were merged. To cluster contigs into MAGs, manual binning and refinement were performed using “anvi-interactive” with both sequence composition (tetranucleotide frequency) and differential coverage across all samples, following previously described approaches to generate high-quality MAGs (90, 91). For coassemblies derived from whole kelp tissue extracts, there was one large bin in each assembly consisting of genomic reads from the host kelp, which was clearly differentiated from bacterial bins based on GC content and differential coverage. Further, BLAST searching revealed that sequences from these bins had a high percent identity match to other brown alga and eukaryotic genomes. To remove host reads from further analyses, the bin containing kelp genomic reads was deselected from the final bin collection in anvi’o. While binning, microbial taxonomy was estimated within anvi’o using “anvi-run-scg-taxonomy,” which searches single-copy genes from each genome against the Genome Taxonomy Database (GTDB). After binning each assembly using “anvi-interactive,” bacterial bins were individually inspected using “anvi-refine,” where they were checked for contaminating contig clusters with dissimilar GC content and differential coverage. Final bin collections were checked for completeness and contamination (also referred to as redundancy) using “anvi-summarize.”

The final MAG collection, curated using “anvi-rename-bins,” contains MAGs with >50% completion and <10% redundancy. Final MAGs were named based on the following scheme: the prefix g1 to g4 corresponds to the metagenome assembly from which it was binned (Table S1), while the numbers distinguish unique MAGs within each coassembly (e.g., g1_MAG_00001). In total, we assembled 28 high-quality MAGs (>90% completion, <5% contamination) and 51 medium-quality MAGs (>50% completion and <10% contamination), according to the genomic standards in reference 92 (Table S2).

### Taxonomic assignment and curation of a nonredundant MAG data set.

To identify highly similar MAGs and pick representative genomes out of the redundant MAGs, the command “anvi-dereplicate-genomes” was used to dereplicate the final MAG collection at 99% average nucleotide identity (ANI) using PyANI (93). Out of the 79 MAGs, 13 were redundant, generating a final data set of 66 nonredundant (or unique) MAGs (Table S2). Representative MAGs with the highest completion combined with the lowest redundancy were selected. Final analyses were conducted with the nonredundant MAG data set. Taxonomy was assigned to each MAG using GTDB-Tk (v1.3.0) (94), which uses a set of 120 concatenated bacterial gene markers to place MAGs in a reference tree based on the Genome Taxonomy Database (release 95) (95), using both FastANI (35) and pplacer (96). MAGs were exported from anvi’o for GTDB-Tk classification using anvi-summarize. The final maximum-likelihood phylogenetic tree was inferred using the IQ-TREE software (97) with the alignment of 120 concatenated bacterial gene markers from GTDB-Tk. Using IQ-TREE, ModelFinder (98) was first implemented to select the best-fit nucleotide substitution model (LG+F+R6), and bootstrap support values were obtained with 1,000 bootstrap replicates.

We determined the detection and abundance of nonredundant MAGs across metagenome samples using “anvi-summarize.” Detection data were visualized using “anvi-interactive” with the minimum detection set to 0.70, indicating that a genome is considered present in a sample if at least 70% of nucleotides in the genome are covered by at least one short read from that sample. We visualized the abundance of nonredundant MAGs within samples using the R packages phyloseq (version 1.30) (99) and ggplot2 (version 3.3.0) (100).

### Pangenomic analysis of *Granulosicoccus*.

The term “pangenome” describes the genes present in all genomes of a given species, which can be subdivided into core genes that are shared by all members of a given species, and accessory genes, present in some but not all genomes of a given species (101). In this study, we generated 8 novel MAGs of *Granulosicoccus*, including 6 of high quality (>90% completion; Table S2). We generated a pangenome with these 8 MAGs, along with two publicly available *Granulosicoccus* genomes: the complete isolate genome of Granulosicoccus antarcticus type strain IMCC3135 (37) and *Granulosicoccus* MAG 002746645, a MAG assembled from a bottlenose dolphin’s mouth (38). To compare the average nucleotide identities (ANIs) among *Granulosicoccus* genomes, we used “anvi-compute-genome-similarity” with PyANI (93). We used anvi’o to analyze and visualize the pangenome with “anvi-display-pan.” First, we made a database of all 10 genomes using “anvi-gen-genomes-storage” and generated the pangenome using “anvi-pan-genome” (minbit set to 0.5 and mcl-inflation set to 10), which uses NCBI’s BLAST to quantify sequence similarity within and between genomes. To obtain functional gene annotations and amino acid sequences from all genes within each *Granulosicoccus* genome, we used “anvi-summarize.” Finally, to search for KEGG metabolic pathways present in the pangenome, we used “anvi-estimate-metabolism” with an E value threshold of 1e−05 and a bitscore fraction of 0.5.

### Functional gene presence-absence across all MAGs.

We constructed a database of annotated genes (COG, KEGG, and Pfam) in each MAG based on anvi’o-generated tables and quantified the number of genes annotated with a given function in each MAG using custom python scripts (for example, see https://github.com/kkmiranda/PNWMetagenomes/blob/main/microbial_metabolisms/finalNitro.py). We searched for genes involved in dissolved organic matter (DOM) transport (list adapted from reference 31), nitrogen metabolism and transformation (56 genes, including those involved in nitrogen fixation, assimilatory and dissimilatory nitrate reduction, denitrification, nitrification, comammox, and urea hydrolysis), and vitamin B_12_ synthesis (list adapted from references 76 and 102) and genes encoding laminarin and alginate metabolism enzymes. E values are the expected number of false-positive hits for a gene annotation, adjusted to the sequence database size. We used E value cutoffs of 1e−20 for KEGG annotations and 1e−50 for COG annotations. We visualized the presence-absence of functional genes across MAGs with heatmaps generated using “anvi-interactive,” where the python-generated functional gene tables were imported as additional layers.

### Phylogenetic analysis of photosystem II (*pufLM*) genes.

To validate the presence of genes for aerobic anoxygenic phototrophy (AAP) in *Granulosicoccus* MAGs and determine their relationship to other known AAP bacteria, we extracted amino acid sequences for the *pufL* and *pufM* photosystem II reaction center genes from each MAG using “anvi-summarize.” We acquired a reference database of 167 *pufL* and *pufM* sequences from reference 44 including 99 belonging to the *Alphaproteobacteria*, 14 to the *Betaproteobacteria*, and 49 to the *Gammaproteobacteria* and 5 from the phylum *Chloroflexi* that were used as an outgroup, as in reference 72. Concatenated *pufLM* sequences were aligned using MAFFT v7.309 (103). A maximum-likelihood phylogeny was inferred using IQ-TREE (97), with ModelFinder (98) to select the best-fit nucleotide substitution model, partitioned for *pufL* and *pufM* genes. Model selection resulted in the best-fit model of LG+F+I+G4 across both genes, and bootstrap support values were obtained with 1,000 bootstrap replicates.

### Data availability.

In addition to the code available on GitHub, the final MAG database files generated in anvi’o are available on the Figshare repository: https://figshare.com/s/84c036dc253a5dd1b1b9. Metagenomic sequence data and bacterial genomes are available at the NCBI’s Sequence Read Archive under accession no. PRJNA783443, including the 7 metagenomes (BioSample accession numbers SAMN23429948 to SAMN23429954) and the 66 nonredundant metagenome-assembled genomes (SAMN27917399 to SAMN27917464).
