# Supplementary figures and images for: Functional Insights into the Kelp Microbiome from Metagenome-Assembled Genomes
Source: mSystems. 2022 Jun 1;7(3):e01422-21. doi: 10.1128/msystems.01422-21 (PMC9238374; doi:10.1128/msystems.01422-21)

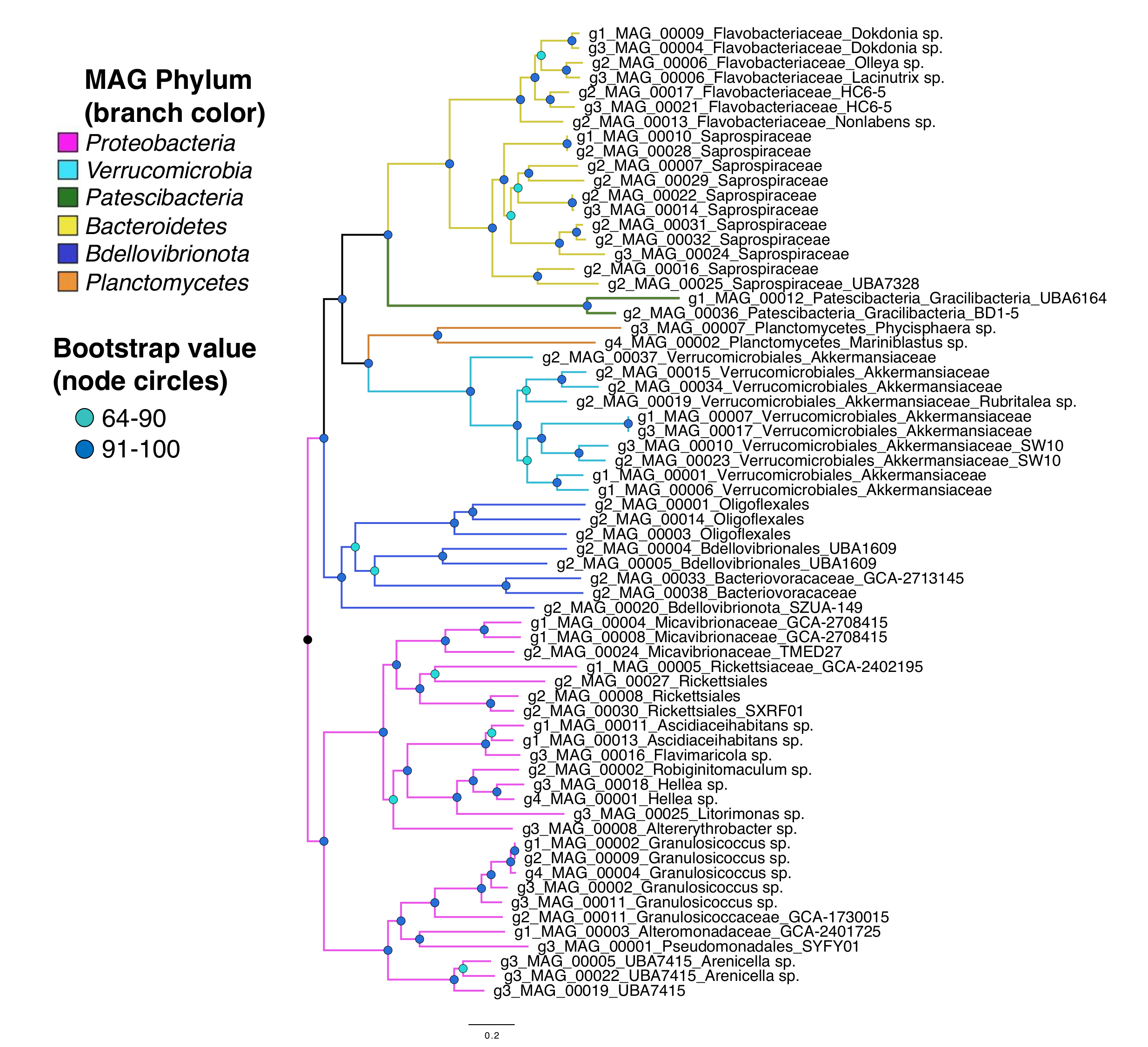

Supplement: FIG S1 [file msystems.01422-21-s0008.tif]

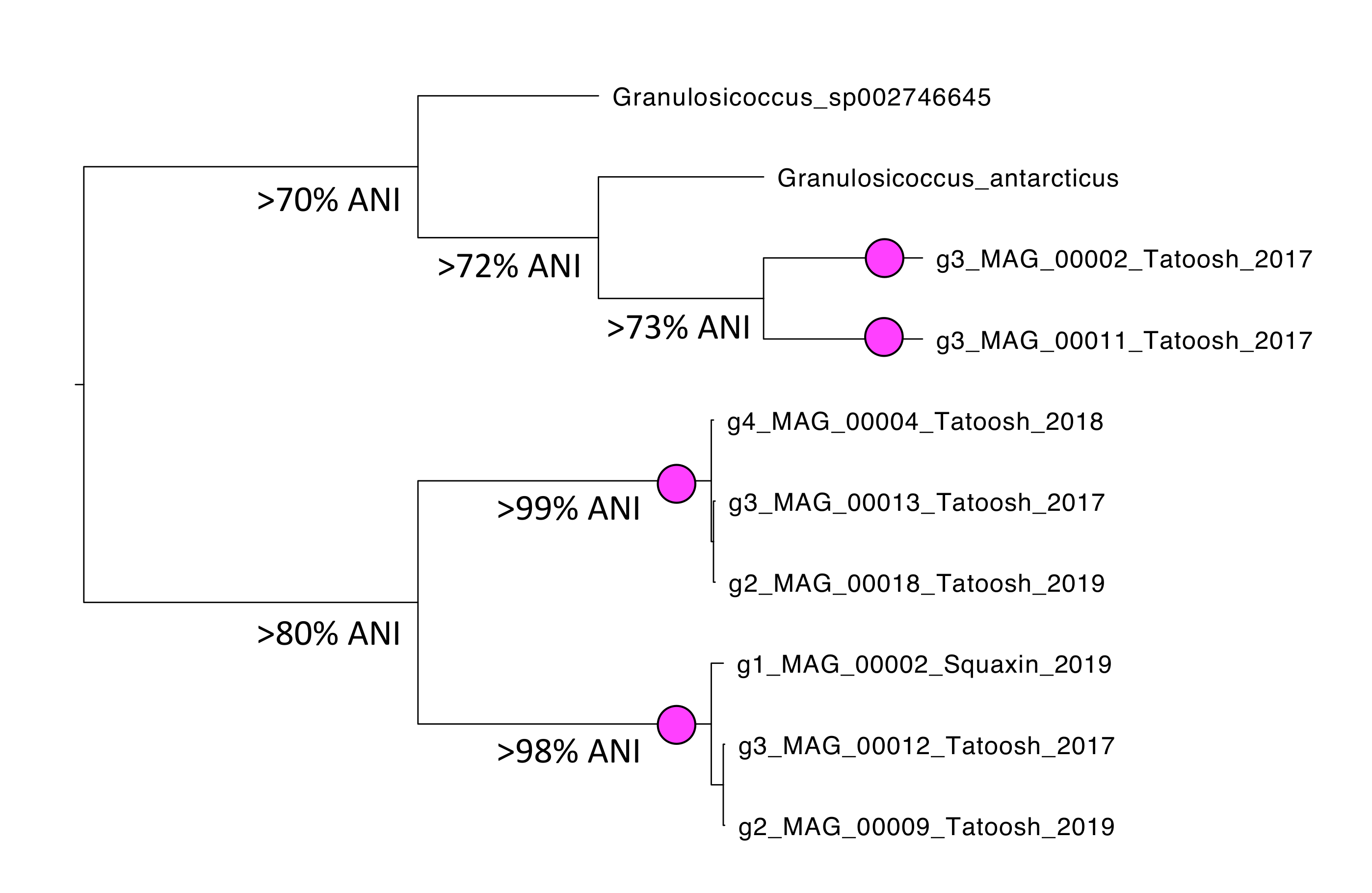

Supplement: FIG S2 [file msystems.01422-21-s0009.tif]

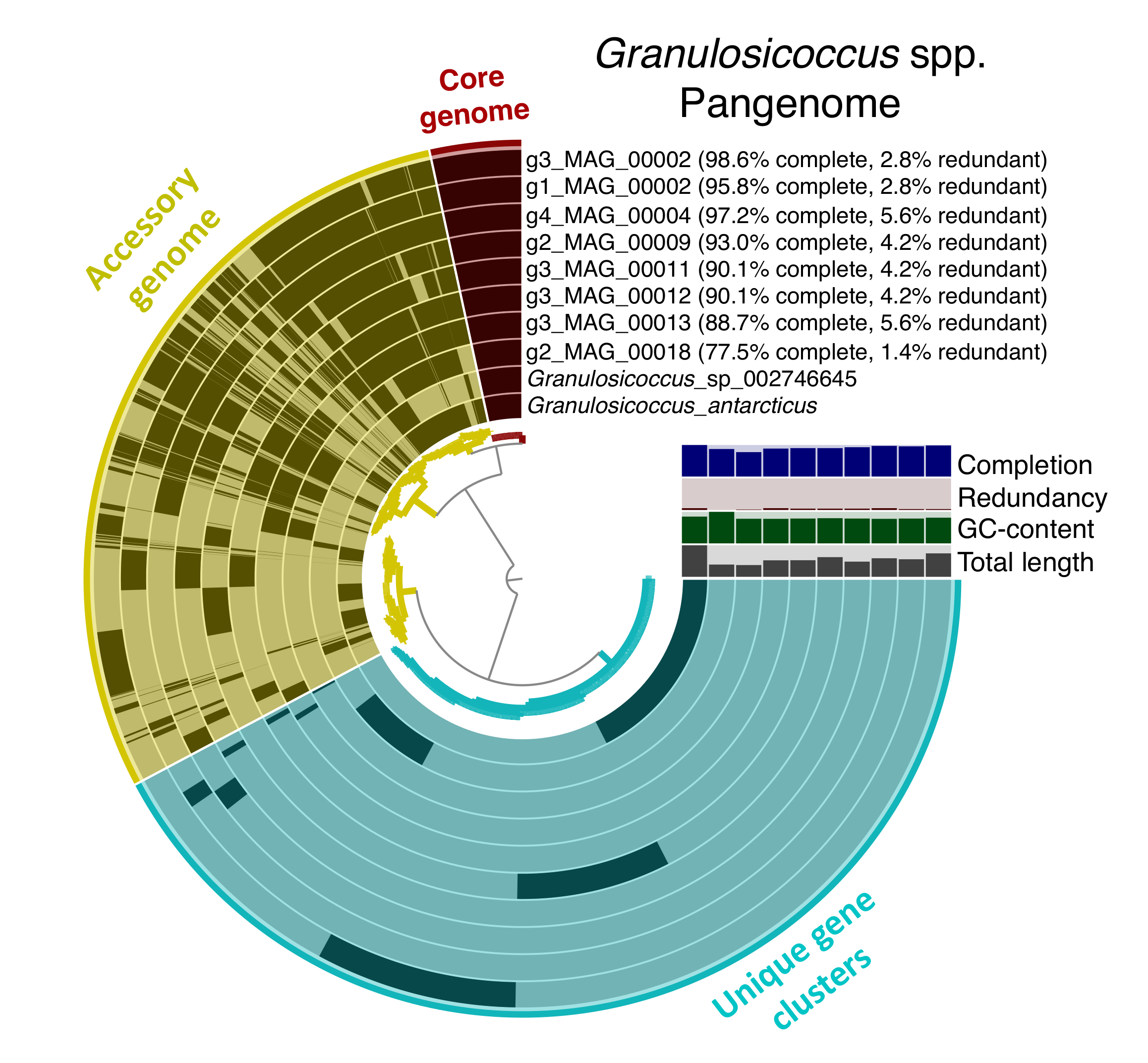

Supplement: FIG S3 [file msystems.01422-21-s0010.tif]
